# New York State Veterinary Medical Society Notice

**Published:** 1893-12

**Authors:** N. P. Hinkley

**Affiliations:** Secretary; No. 395 Ellicott Street, Buffalo, N. Y.


### NOTICE TO THE VETERINARIANS OF THE STATE OF NEW YORK.

The New York State Veterinary Medical Society will hold its Fourth Annual Meeting at the Assembly Room of the Vanderbilt House, Syracuse, N. Y., on Tuesday and Wednesday, January 9th and 10th, 1894. The meeting will commence at 11 o'clock A. M., January 9th. This meeting will be the last one to be held in the month of January, as, according to the revised By-Laws adopted by the Society at last meeting held in Buffalo. Oct. 11 and 12, 1893, hereafter the Society will hold its meetings during the month of September, each year, and the place of holding said meeting will be decided by a vote of the majority of the members present at the close of the Annual Meeting. The January meeting will be an interesting one to all veterinarians. There will be several papers read and discussed. The business portion of the meeting, consisting of Election of Officers, Reports of Secretaries, Treasurer and Committees, and Auditing of Accounts, will be made as short as possibie, thereby giving more time to the reading and discussing of papers The names of those writing papers and the subjects will be printed in the January issue of the JOURNAL. All desiring to apply for membership should address the Secretary at once. Printed notices will be mailed to all veterinarians.

Respectfully,

No. 395 Ellicott Street, N. P. HINKLEY, D. V. S.,
Buffalo, N. Y. *Secretary.*

